# The novel inhaled dual PDE3 and PDE4 inhibitor ensifentrine for the treatment of COPD: A systematic review and meta-analysis protocol on trough FEV_1_ and exacerbation according to PRISMA statement

**DOI:** 10.1016/j.crphar.2024.100195

**Published:** 2024-07-06

**Authors:** Luigino Calzetta, Mario Cazzola, Shima Gholamalishahi, Paola Rogliani

**Affiliations:** a Department of Medicine and Surgery, Respiratory Disease and Lung Function Unit, University of Parma, Parma, Italy; b Department of Experimental Medicine, Unit of Respiratory Medicine, University of Rome “Tor Vergata”, Rome, Italy

## Abstract

The investigation of ensifentrine, an inhaled dual phosphodiesterase (PDE)3 and PDE4 inhibitor, for chronic obstructive pulmonary disease (COPD) maintenance therapy presents a significant clinical interest. Despite promising results from recent Phase III trials, a comprehensive synthesis of its therapeutic efficacy in COPD is lacking. This protocol outlines the first registered systematic review and meta-analysis in PROSPERO to assess the impact of ensifentrine on trough forced expiratory volume in the 1st second (FEV_1_) and acute exacerbations of COPD. By conducting a rigorous literature search and employing solid methodologies, this endeavour aims to provide robust evidence on the real efficacy of ensifentrine. Anticipated outcomes include a significant improvement in trough FEV_1_ and a reduction in AECOPD risk among ensifentrine-treated patients compared to controls, corroborating its bronchodilator and anti-inflammatory properties. The meta-analysis expects to reveal consistent results across different trials, enhancing confidence in the findings. Additionally, subgroup analyses may unveil factors influencing the efficacy of ensifentrine, guiding optimal therapeutic strategies. Overall, this protocol holds the potential to inform clinical practice and regulatory decisions, positioning ensifentrine as a valuable addition to COPD management.

## 1 Introduction

**Fig. 1:**
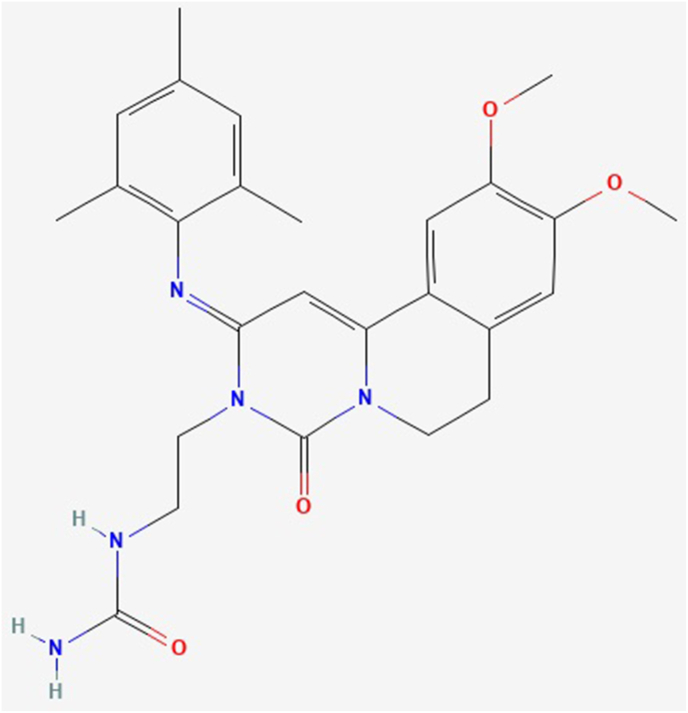
Chemical structure of ensifentrine, formerly known as RPL554.

The novel inhaled dual phosphodiesterase (PDE)3 and PDE4 inhibitor ensifentrine (CAS 298680-25-8, molecular formula C_26_H_31_N_5_O_4_, molecular weight 477.6 g/mol) formerly known as RPL554 (Calzetta et al., 2013a, 2013b; Franciosi et al., 2013), is currently under investigation by the Food and Drug Administration (FDA) for the maintenance therapy of chronic obstructive pulmonary disease (COPD) (Verona Pharma). The 2D structure of ensifentrine is shown in Fig. 1 and the animated 3D structure is available in the supplementary multimedia file (Ensifentrine | C26H31N5O4 | CID). The main pharmacological characteristics of ensifentrine in human airways and inflammatory cells are reported in Table 1, according to current pharmacological evidence (Calzetta et al., 2013b, 2015; Boswell-Smith et al., 2006).

**Table 1:** Pharmacological characteristics of ensifentrine in human inflammatory cells and human medium and small airways.

Pharmacological variables	Experimental results in human inflammatory cells and airways						References
	PDE3 in purified human platelets	PDE4 in purified human neutrophils	Ratio PDE3/PDE4	TNF-α production from human monocytes stimulated by LPS	Proliferation of human mononuclear cells stimulated by PHA	/	Boswell-Smith et al. (2006)
IC_50_	0.4 nM	1479 nM	3440	0.52 μM	0.46 μM	/	
	Medium human airways sub-maximally pre-contracted by ACh	Medium human airways sub-maximally pre-contracted by His	Medium human airways contracted by EFS	Small human airways sub-maximally pre-contracted by CCh	/	/	(Calzetta et al., 2013b, 2015)
E_max_	100%	100%	100%	100%	/	/	
Hill slope	2.27	0.88	/	/	/	/	
EC_50_	21.2 μM	12.9 μM	5.4 μM	1.1 μM	/	/	
pEC_50_	4.67	4.89	5.27	5.96	/	/	
T_1/2_	/	/	24 min	/	/	/	
Duration of action	/	/	>4 h	/	/	/	
	Ensifentrine plus salbutamol in medium huma airways sub-maximally pre-contracted by ACh	Ensifentrine plus atropine in medium human airways sub-maximally pre-contracted by ACh	Ensifentrine plus glycopyrronium in medium human airways sub-maximally pre-contracted by ACh	Ensifentrine plus glycopyrronium in medium human airways sub-maximally pre-contracted by His	Ensifentrine plus glycopyrronium in medium human airways contracted by EFS	Ensifentrine plus glycopyrronium in small human airways sub-maximally pre-contracted by CCh	
Significant synergistic relaxant effect	No	Yes	Yes	Yes	Yes	Yes	
Maximal delta effect (observed – expected)	/	54%	46%	46%	36%	28%	
Time to maximal synergistic interaction	/	/	/	/	2 h	30 min	
Duration of action	/	/	/	/	>6 h	/	

data not available; ACh: acetylcholine; CCh: carbachol; EC_50_: the concentration of producing half-E_max_; EFS: electrical field stimulation; E_max_: maximal effect; His: histamine; IC50: the concentration of an antagonist that reduces the response to an agonist by 50%; LPS: lipopolysaccharide; PDE: phosphodiesterase inhibitor; pEC_50_: negative logarithm of EC_50_; PHA: phytohemagglutinin; T1/2: onset of action.

Recent clinical evidence from Phase III randomized controlled trials (RCTs) has yielded promising and intriguing results when administered via nebulization (NCT04542057, NCT04535986) (Anzueto et al., 2023). Further evidence supports significant improvements in lung functions in COPD patients when ensifentrine is administered via dry powder inhaler (DPI) and pressurized metered-dose inhaler (pMDI), although these data originate from Phase II RCTs (NCT04027439, NCT04091360) (Rheault et al., 2020; Rheault et al., 2021).

According to its pharmacological characteristics, ensifentrine combines both bronchodilator and anti-inflammatory properties, distinguishing this molecule from any other COPD treatment that typically targets only one of these outcomes (Calzetta et al., 2024; Cazzola et al., 2023). Given the significant likelihood of its approval in the near future, ensifentrine could represent a major breakthrough. Notably, it has been over a decade since a drug with a novel mechanism of action was approved for the maintenance treatment of COPD (Calzetta et al., 2023, 2024).

The measurement of trough forced expiratory flow in the 1st second (FEV_1_) is pivotal in COPD patients as it represents a global marker for pathophysiological changes, is strictly related to patient-reported outcomes, and is used by regulatory agencies for the drug approval process (Donohue et al., 2018; Westwood et al., 2011; Jones et al., 2011; Jones and Agusti, 2006). Nevertheless, trough FEV_1_ has never been investigated as a primary endpoint in the RCTs constituting the pipeline development of ensifentrine in COPD (Pipeline - Verona Pharma). Generally, FEV_1_ area under the curve at 0–12 h (AUC_0–12h_) has been identified as a primary outcome, relegating trough FEV_1_ to a less important secondary outcome (NCT04542057, NCT04535986, NCT04027439, NCT04091360).

This represents an important issue for development strategy of ensifentrine, particularly given that the minimal clinically important difference (MCID) value, which regulatory authorities use to interpret drug efficacy in COPD trials, is available for trough FEV_1_ but not for FEV_1_ AUC_0–12h_ (Jones et al., 2014; Cazzola et al., 2008; Manzetti et al., 2023).

Therefore, considering the current background and scientific evidence, the primary aim of this paper is to provide a protocol for conducting a high-quality systematic review and meta-analysis on the effect of ensifentrine on trough FEV_1_ in COPD patients. In line with the importance of pharmacological therapy for the prevention of AECOPD (MacLeod et al., 2021; Wedzicha et al., 2017), this systematic review and meta-analysis also examine this outcome.

## 2 Materials and methods

### 2.1 Search strategy

The protocol of this systematic review and meta-analysis is registered in International Prospective Register of Systematic Reviews (PROSPERO; registration number: CRD42023478153) in accordance with the Preferred Reporting Items for Systematic Reviews and Meta-Analyses Protocols (PRISMA-P) (Moher et al., 2015).

Two reviewers perform a comprehensive literature search for RCTs evaluating the effects of ensifentrine in patients with COPD. The PICO (patient problem, intervention, comparison, and outcome framework) is used to develop the literature search strategy, as previously described (Schardt et al., 2007). Specifically, the patient problem includes subject affected by COPD; the intervention is ensifentrine, administered either as a single agent or added on top of any maintenance COPD therapy; the comparison includes placebo (PCB); the outcomes are trough FEV_1_ and acute exacerbation of COPD (AECOPD).

The terms “ensifentrine” OR “RPL554” are searched in ClinicalTrials.gov, Cochrane Central Register of Controlled Trials (CENTRAL), EMBASE, EU Clinical Trials Register, and MEDLINE via PubMed without language restrictions.

### 2.2 Study selection

RCTs involving patients with COPD treated with ensifentrine are included in this meta-analysis, while open-label studies are excluded. Two reviewers independently check the relevant studies identified from literature searches obtained from the previously mentioned databases. The titles and abstracts of all the records identified in initial research are reviewed, and then a list of full text articles to be assessed for selection is defined. RCTs reporting data concerning the impact of ensifentrine on patients suffering from COPD are selected, without limitations for study duration. Full text articles not reporting data from RCTs, and/or performed in non-COPD patients, and/or not reporting trough FEV_1_ and/or acute AECOPD in the MEDLINE are excluded. The studies are selected in accordance with the previously mentioned criteria, and any difference in opinion about eligibility are resolved by consensus.

### 2.3 Quality of studies, risk of bias, and evidence profile

The summary of the risk of bias for each included RCT is analysed via the Jadad score (Halpern and Douglas, 2005) and Cochrane Risk of Bias 2 (RoB2) (Guyatt et al., 2011). The Jadad score, with a scale of 1–5 (score of 5 being the best quality), is used to assess the quality of the RCTs regarding the likelihood of biases related to randomization, double blinding, withdrawals, and dropouts (Jadad et al., 1996). Studies are considered of low quality at Jadad score <3, of medium quality at Jadad score = 3, and of high quality at Jadad score >3 (Halpern and Douglas, 2005). The weighted assessment of the risk of bias is analysed via the Cochrane RoB 2 (Guyatt et al., 2011).

Funnel plot and Egger's test are performed to assess the origin and risk of publication bias for the primary endpoint if ≥ 10 studies are included in the meta-analysis (Page et al., 2019). For Egger's test, the following regression equation is applied: SND = a + b × precision, where SND represents the standard normal deviation (treatment effect divided by its standard error [SE]), and precision represents the reciprocal of the SE. Evidence of asymmetry from Egger's test is considered to be significant for P < 0.01 and the graphical representation of 90% confidence bands is reported (Sterne et al., 2000; Sterne and Egger, 2001; Egger et al., 1997).

The quality of the evidence is assessed for the primary endpoint in accordance with the Grading of Recommendations Assessment, Development, and Evaluation (GRADE) system, indicating ++++ for high quality of evidence, +++ for moderate quality of evidence, ++ for low quality of evidence, and + for very low quality of evidence (Guyatt et al., 2011). Two reviewers independently assess the quality of individual studies and any difference in opinion is resolved by consensus.

### 2.4 Data extraction

The data sourced from RCTs are extracted and verified through a comprehensive examination of published papers. The extraction process involves several studies and patients characteristics, including the study duration, inhaler device, age, gender, smoking habits, FEV_1_, AECOPD, and items to calculate the Jadad score. Data extraction is conducted independently by two reviewers and any and any discrepancy or inconsistency encountered during the extraction process is resolved by consensus. Due to the complexity of this meta-analysis, data are extracted in accordance with the recommendations provided by the Cochrane Handbook for Systematic Reviews of Interventions (Higgins and Green, 2011).

### 2.5 Endpoints

The primary endpoint is the effect of ensifentrine on the change in trough FEV_1_ in COPD patients; the secondary endpoint is the effect of ensifentrine on the risk of moderate or severe AECOPD.

### 2.6 Data analysis

A pairwise meta-analysis is performed to quantify the effect of ensifentrine on the primary and secondary endpoints. The obtained results are expressed as mean difference (MD) or relative risk (RR), according to the analysed variables, with 95% confidence interval (95%CI).

Generally, data from RCTs are extracted from a series of studies performed by researchers operating independently, and therefore a common effect size cannot be assumed. Thus, the binary derSimonian-Laird random-effects model is used to balance the study weights and correctly assess the effect estimates and relative 95%CI (Calzetta et al., 2016).

The test for heterogeneity (I^2^) is performed to quantify the between-study dissimilarity, as previously reported (Wallace et al., 2012), and sensitivity analysis is carried out to identify the studies that introduce substantial and significant levels of heterogeneity (I^2^>50%, P < 0.05) in the quantitative synthesis (Higgins et al., 2003). If necessary, meta-regression analysis is performed for the primary endpoint to identify potential effect modifier that could alter the efficacy of ensifentrine on trough FEV_1_ (Cazzola et al., 2017).

Subgroup analyses are carried out with respect to the specific maintenance COPD therapies to which ensifentrine is added and the dose of ensifentrine under evaluation by the FDA.

### 2.7 Software and statistical significance

GraphReader is used to extract data from the figures, when necessary (http://www.graphreader.com/); OpenMeta-Analyst (version 12.11.14, Wallace et al., Tufts University, Boston, MA, USA) (Wallace et al., 2012) software is used to perform the pairwise meta-analysis; GraphPad Prism (version 7.0a, GraphPad Software Inc., San Diego, CA, USA) software is used to graph the data; GRADEpro GDT software (online version available from gradepro.org, McMaster University and Evidence Prime Inc., Hamilton, ON, Canada) is used to assess the quality of evidence (Guyatt et al., 2011), and the robvis visualization software (online version available from mcguinlu.shinyapps.io/robvis/, McGuinness et al., University of Bristol, Bristol, UK) is used to apply the RoB 2 tool (Sterne et al., 2019; McGuinness and Higgins, 2021). The statistical significance of the effect estimates resulting from the pairwise meta-analysis is assessed for P < 0.05.

## 3 Expected results

To date, there exists no published systematic review or meta-analysis offering a quantitative synthesis of the therapeutic efficacy of ensifentrine in COPD, making this protocol the first formal endeavour registered in PROSPERO to specifically investigate trough FEV_1_ and AECOPD.

This protocol allows performing a high-quality systematic review and meta-analysis on the effect of enisfentrine on trough FEV_1_ and AECOPD in COPD patients. The primary endpoint is expected to demonstrate a significant improvement in trough FEV_1_ among COPD patients treated with ensifentrine, administered either as a single agent or added on top of any maintenance COPD therapy, compared to those receiving PCB. This improvement should provide robust evidence of the efficacy of ensifentrine as a bronchodilator, potentially meeting or exceeding the MCID value for trough FEV_1_. Additionally, the secondary endpoint is likely to indicate a reduction in the risk of moderate or severe AECOPD in patients treated with ensifentrine, highlighting the potential role of ensifentrine in not only improving lung function but also in reducing the risk of AECOPD according to its anti-inflammatory activity.

The meta-analysis is expected to show consistent results across various RCTs, suggesting that the observed effects of ensifentrine are reliable and not significantly influenced by study-specific factors. Given the rigorous methodology outlined, including the use of tools such as the Jadad score and Cochrane Risk of Bias 2 tool, the quality of the evidence is anticipated to be high, enhancing confidence in the findings and supporting the robustness of the conclusions drawn from the meta-analysis.

The test for I^2^, which indicates between-study variability, provides insight into the level of generalizability across different patient populations and study conditions. Funnel plots and Egger's test are expected to reveal the risk of publication bias, ensuring that the results are not skewed by selective reporting or other biases. Sensitivity analyses should further corroborate the stability of the findings.

Subgroup analyses might reveal differential effects of ensifentrine based on factors such as the specific maintenance COPD therapies to which ensifentrine is added and the dose of ensifentrine, providing insights into optimal use conditions and dosing regimens. Overall, the findings are expected to have significant implications for clinical practice by supporting the use of ensifentrine as a novel effective treatment for COPD. This will help clinicians make informed decisions about incorporating it into treatment regimens, tailoring therapy to meet individual patient needs, and optimizing its use. Additionally, the results may aid regulatory agencies like the FDA in making informed decisions regarding the approval of ensifentrine for COPD maintenance therapy by providing the necessary data to demonstrate the drug's efficacy. Furthermore, the results could inform the development or update of treatment guidelines for COPD by regulatory bodies and medical associations, including ensifentrine as a recommended therapy.

Ultimately, the systematic review and meta-analysis are expected to provide comprehensive and high-quality evidence on the efficacy of ensifentrine, positioning it as a valuable addition to the therapeutic armamentarium for COPD.

## CRediT authorship contribution statement

**Luigino Calzetta:** Conceptualization, Methodology, Software, Validation, Investigation, Resources, Writing – original draft, Writing – review & editing, Supervision, Project administration, Funding acquisition. **Mario Cazzola:** Investigation, Writing – original draft, Writing – review & editing. **Shima Gholamalishahi:** Methodology, Investigation, Writing – original draft, Writing – review & editing. **Paola Rogliani:** Conceptualization, Methodology, Software, Validation, Investigation, Resources, Writing – original draft, Writing – review & editing, Supervision, Project administration, Funding acquisition.

## Declaration of competing interest

No conflict of interest.

## Data Availability

No data was used for the research described in the article.

## References

[bib1] Anzueto A., Barjaktarevic I.Z., Siler T.M., Rheault T., Bengtsson T., Rickard K., Sciurba F. (2023). Ensifentrine, a novel PDE3 and PDE4 inhibitor for the treatment of COPD: randomized, double-blind, placebo-controlled, multicenter, Phase III trials (The ENHANCE Trials). Am. J. Respir. Crit. Care Med..

[bib2] Boswell-Smith V., Spina D., Oxford A.W., Comer M.B., Seeds E.A., Page C.P. (2006). The pharmacology of two novel long-acting phosphodiesterase 3/4 inhibitors, RPL554 [9,10-dimethoxy-2(2,4,6-trimethylphenylimino)-3-(N-carbamoyl-2- aminoethyl)-3,4,6,7-tetrahydro-2H-pyrimido[6,1-a]isoquinolin-4-one] and RPL565 [6,7-dihydro-2-(2,6-diisoprop. J. Pharmacol. Exp. Therapeut..

[bib3] Calzetta L., Page C.P., Spina D., Cazzola M., Rogliani P., Facciolo F., Matera M.G. (2013). Effect of the mixed phosphodiesterase 3/4 inhibitor RPL554 on human isolated bronchial smooth muscle tone. J. Pharmacol. Exp. Therapeut..

[bib4] Calzetta L., Page C.P., Spina D., Cazzola M., Rogliani P., Facciolo F., Matera M.G. (2013). Effect of the mixed phosphodiesterase 3/4 inhibitor RPL554 on human isolated bronchial smooth muscle tone. J. Pharmacol. Exp. Therapeut..

[bib5] Calzetta L., Cazzola M., Page C.P., Rogliani P., Facciolo F., Matera M.G. (2015). Pharmacological characterization of the interaction between the dual phosphodiesterase (PDE) 3/4 inhibitor RPL554 and glycopyrronium on human isolated bronchi and small airways. Pulm. Pharmacol. Ther..

[bib6] Calzetta L., Rogliani P., Matera M.G., Cazzola M. (2016). A systematic review with meta-analysis of dual bronchodilation with LAMA/LABA for the treatment of stable COPD. Chest.

[bib7] Calzetta L., Pistocchini E., Chetta A., Rogliani P., Cazzola M. (2023). Experimental drugs in clinical trials for COPD: artificial intelligence via machine learning approach to predict the successful advance from early-stage development to approval. Expet Opin. Invest. Drugs.

[bib8] Calzetta L., Cazzola M., Rogliani P. (2024). Pharmacological interpretation of the efficacy of ensifentrine in chronic obstructive pulmonary disease: insights from ENHANCE trials. Am. J. Respir. Crit. Care Med..

[bib9] Cazzola M., MacNee W., Martinez F.J., Rabe K.F., Franciosi L.G., Barnes P.J., Brusasco V., Burge P.S., Calverley P.M.A., Celli B.R., Jones P.W., Mahler D.A., Make B., Miravitlles M., Page C.P., Palange P., Parr D., Pistolesi M., Rennard S.I., Rutten-van Mölken M.P., Stockley R., Sullivan S.D., Wedzicha J.A., Wouters E.F. (2008). Outcomes for COPD pharmacological trials: from lung function to biomarkers. Eur. Respir. J..

[bib10] Cazzola M., Rogliani P., Calzetta L., Hanania N.A., Matera M.G. (2017). Impact of mucolytic agents on COPD exacerbations: a pair-wise and network meta-analysis. COPD.

[bib11] Cazzola M., Page C., Calzetta L., Singh D., Rogliani P., Matera M.G. (2023). What role will ensifentrine play in the future treatment of chronic obstructive pulmonary disease patients? Implications from recent clinical trials. Immunotherapy.

[bib12] Donohue J.F., Jones P.W., Bartels C., Marvel J., D'Andrea P., Banerji D., Morris D.G., Patalano F., Fogel R. (2018). Correlations between FEV1 and patient-reported outcomes: a pooled analysis of 23 clinical trials in patients with chronic obstructive pulmonary disease. Pulm. Pharmacol. Ther..

[bib13] Egger M., Davey Smith G., Schneider M., Minder C. (1997). Bias in meta-analysis detected by a simple, graphical test. BMJ.

[bib14] Ensifentrine | C26H31N5O4 | CID 9934746 - PubChem, (n.d.). https://pubchem.ncbi.nlm.nih.gov/compound/Ensifentrine (accessed June 18, 2024).

[bib15] Franciosi L.G., Diamant Z., Banner K.H., Zuiker R., Morelli N., Kamerling I.M.C., de Kam M.L., Burggraaf J., Cohen A.F., Cazzola M., Calzetta L., Singh D., Spina D., Walker M.J.A., Page C.P. (2013). Efficacy and safety of RPL554, a dual PDE3 and PDE4 inhibitor, in healthy volunteers and in patients with asthma or chronic obstructive pulmonary disease: findings from four clinical trials. Lancet Respir. Med..

[bib16] Guyatt G., Oxman A.D., Akl E.A., Kunz R., Vist G., Brozek J., Norris S., Falck-Ytter Y., Glasziou P., DeBeer H., Jaeschke R., Rind D., Meerpohl J., Dahm P., Schünemann H.J. (2011). GRADE guidelines: 1. Introduction-GRADE evidence profiles and summary of findings tables. J. Clin. Epidemiol..

[bib17] Halpern S.H., Douglas M.J. (2005).

[bib18] Higgins J.P.T., Green S. (2011).

[bib19] Higgins J.P.T., Thompson S.G., Deeks J.J., Altman D.G. (2003). Measuring inconsistency in meta-analyses. Bmj.

[bib20] Jadad A.R., Moore R.A., Carroll D., Jenkinson C., Reynolds D.J., Gavaghan D.J., McQuay H.J. (1996). Assessing the quality of reports of randomized clinical trials: is blinding necessary?. Contr. Clin. Trials.

[bib21] Jones P.W., Agusti A.G.N. (2006). Outcomes and markers in the assessment of chronic obstructive pulmonary disease. Eur. Respir. J..

[bib22] Jones P.W., Donohue J.F., Nedelman J., Pascoe S., Pinault G., Lassen C. (2011). Correlating changes in lung function with patient outcomes in chronic obstructive pulmonary disease: a pooled analysis. Respir. Res..

[bib23] Jones P.W., Beeh K.M., Chapman K.R., Decramer M., Mahler D.A., Wedzicha J.A. (2014). Minimal clinically important differences in pharmacological trials. Am. J. Respir. Crit. Care Med..

[bib24] MacLeod M., Papi A., Contoli M., Beghé B., Celli B.R., Wedzicha J.A., Fabbri L.M. (2021). Chronic obstructive pulmonary disease exacerbation fundamentals: diagnosis, treatment, prevention and disease impact. Respirology.

[bib25] Manzetti G.M., Ora J., Sepiacci A., Cazzola M., Rogliani P., Calzetta L. (2023). Clinically important deterioration (CID) and ageing in COPD: a systematic review and meta-regression analysis according to prisma statement. Int. J. COPD.

[bib26] McGuinness L.A., Higgins J.P.T. (2021). Risk-of-bias VISualization (robvis): an R package and Shiny web app for visualizing risk-of-bias assessments. Res. Synth. Methods.

[bib27] Moher D., Shamseer L., Clarke M., Ghersi D., Liberati A., Petticrew M., Shekelle P., Stewart L.A. (2015). Preferred reporting items for systematic review and meta-analysis protocols (PRISMA-P) 2015 statement. Syst. Rev..

[bib28] Page M.J., Higgins J.P.T., Sterne J.A.C. (2019). Assessing risk of bias due to missing results in a synthesis. Cochrane Handbook for Systematic Reviews of Interventions.

[bib29] Pipeline - Verona Pharma, (n.d.). https://www.veronapharma.com/pipeline (accessed May 15, 2024).

[bib30] Rheault T., Rickard K., Boscia J.A., Bengtsson T. (2020). ATS 2020 International Conference American Thoracic Society International Conference Meetings Abstracts.

[bib31] Rheault T., Singh D., Leaker B., Bengtsson T., Rickard K. (2021). ENSIFENTRINE, a dual PDE 3 and 4 inhibitor, provides effective bronchodilation in patients with COPD when administered twice daily over 7 days via PMDI. Chest.

[bib32] Schardt C., Adams M.B., Owens T., Keitz S., Fontelo P. (2007). Utilization of the PICO framework to improve searching PubMed for clinical questions. BMC Med. Inf. Decis. Making.

[bib33] Sterne J.A., Egger M. (2001). Funnel plots for detecting bias in meta-analysis: guidelines on choice of axis. J. Clin. Epidemiol..

[bib34] Sterne J.A., Gavaghan D., Egger M. (2000). Publication and related bias in meta-analysis: power of statistical tests and prevalence in the literature. J. Clin. Epidemiol..

[bib35] Sterne J.A.C., Savovic J., Page M.J., Elbers R.G., Blencowe N.S., Boutron I., Cates C.J., Cheng H.Y., Corbett M.S., Eldridge S.M., Emberson J.R., Hernan M.A., Hopewell S., Hrobjartsson A., Junqueira D.R., Juni P., Kirkham J.J., Lasserson T., Li T., McAleenan A., Reeves B.C., Shepperd S., Shrier I., Stewart L.A., Tilling K., White I.R., Whiting P.F., Higgins J.P.T. (2019). RoB 2: a revised tool for assessing risk of bias in randomised trials. Bmj.

[bib36] Verona Pharma Announces the US FDA has Accepted the New Drug Application Filing for Ensifentrine for the Maintenance Treatment of COPD - Verona Pharma, (n.d.). https://www.veronapharma.com/media/verona-pharma-announces-us-fda-has-accepted-new-drug-application (accessed April 23, 2024).

[bib37] Wallace B.C., Dahabreh I.J., Trikalinos T.A., Lau J., Trow P., Schmid C.H. (2012). Closing the gap between methodologists and end-users: R as a computational back-end. J. Stat. Software.

[bib38] Wedzicha J.A., Calverley P.M.A., Albert R.K., Anzueto A., Criner G.J., Hurst J.R., Miravitlles M., Papi A., Rabe K.F., Rigau D., Sliwinski P., Tonia T., Vestbo J., Wilson K.C., Krishnan J.A. (2017). Prevention of COPD exacerbations: a European respiratory society/American thoracic society guideline. Eur. Respir. J..

[bib39] Westwood M., Bourbeau J., Jones P.W., Cerulli A., Capkun-Niggli G., Worthy G. (2011). Relationship between FEV1 change and patient-reported outcomes in randomised trials of inhaled bronchodilators for stable COPD: a systematic review. Respir. Res..

